# Distribution of the Crustal Magnetic Field in Sichuan-Yunnan Region, Southwest China

**DOI:** 10.1155/2014/854769

**Published:** 2014-08-26

**Authors:** Chunhua Bai, Guofa Kang, Guoming Gao

**Affiliations:** Department of Geophysics, Yunnan University, Kunming, Yunnan 650091, China

## Abstract

Based on the new and higher degree geomagnetic model NGDC-720-V3, we have investigated the spatial distribution, the altitude decay characteristics of the crustal magnetic anomaly, the contributions from different wavelength bands to the anomaly, and the relationship among the anomaly, the geological structure, and the geophysical field in Sichuan-Yunnan region of China. It is noted that the most outstanding feature in this area is the strong positive magnetic anomaly in Sichuan Basin, a geologically stable block. Contrasting with this feature, a strong negative anomaly can be seen nearby in Longmen Mountain block, an active block. This contradiction implies a possible relationship between the magnetic field and the geological activity. Completely different feature in magnetic field distribution is seen in the central Yunnan block, another active region, where positive and negative anomalies distribute alternatively, showing a complex magnetic anomaly map. Some fault belts, such as the Longmen Mountain fault, Lijiang-Xiaojinhe fault, and the Red River fault, are the transitional zones of strong and weak or negative and positive anomalies. The corresponding relationship between the magnetic anomaly and the geophysical fields was confirmed.

## 1. Introduction

The crustal magnetic field (or lithospheric field) generated by the remanent magnetization and the induced magnetization in the crust and upper mantle is an important part of the geomagnetic field. It is not only related to the magnetized mineral in its origin, but also closely related to the geological tectonics and the lithospheric evolution [1–3]. Because of differences of crustal rocks magnetization properties and tectonic evolution in separate places, the crustal anomaly has characteristics such as stronger regional feature and complicated distribution. Thus, we should choose regional or local area for better understanding of its distribution characteristics.

Following the advances of satellite magnetic survey and data processing technology, a high degree geomagnetic spherical harmonic model compiled from satellite, marine, aeromagnetic, and ground magnetic surveys can then be evaluated at any desired location to provide the magnetic field components, or the anomaly of the field, and to study their spatial and temporal distribution regularities. At present, complete separation of the main (or core) field and the crustal field has not been achieved in theory. In practice, they overlapped each other in measurements. It can be inferred from the geomagnetic power spectrum with the spherical harmonic degree *n* that *n* ≤ 13 is for the main field, *n* = 14~15 is the transition of the main field and the lithospheric field, and *n* ≥ 16 is for the crustal field [4–6], which provides quantitative basis for the study of the crustal field.

In the past decade, significant advances have been achieved in building high degree spherical harmonic geomagnetic field models, and a great number of models have been established worldwide. The model coefficients have been continuously updated with new measurements data increasing. Among these models, some are constructed from satellite data only; many of them are comprehensive models (CMs) derived from satellite, aeromagnetic, ground, and marine magnetic survey data [7–10]. Furthermore, all available magnetic survey data worldwide have been compiled for more than half a century. The World Digital Magnetic Anomaly Map (WDMAM) was developed through a collaborative effort led by staff at the International Association of Geomagnetism and Aeronomy (IAGA) [11–14]. These models are important and fundamental data for studying the crustal magnetic field.

Based on the NGDC-720-V3 model derived from satellite, airborne, ground, and marine magnetic survey data and constructed by the American National Geophysical Data Center (NGDC) (http://www.ngdc.noaa.gov/geomag/NGDC720/index.html) as discussed by Maus [15], we investigate spatial distribution features of the crustal field in the Sichuan-Yunnan region and seek for the connection between the crustal anomaly and the geological structure, including the corresponding relationship between the transitional zone of strong and weak magnetic anomalies and the fault zone, and characteristics of crustal anomalies in basins, orogens, and fracture zones. We mainly concentrate on the spatial distribution and altitude decaying features of the vertical component Δ*Z* and its gradients and contributions from different wavelength bands to the anomaly. The connection between the anomaly and the crust structure is revealed by comparing with geophysical fields such as the seismic wave velocity anomaly, the gravity anomaly, and the lithospheric thermal structure.

## 2. Geologic Background

**Figure 1:**
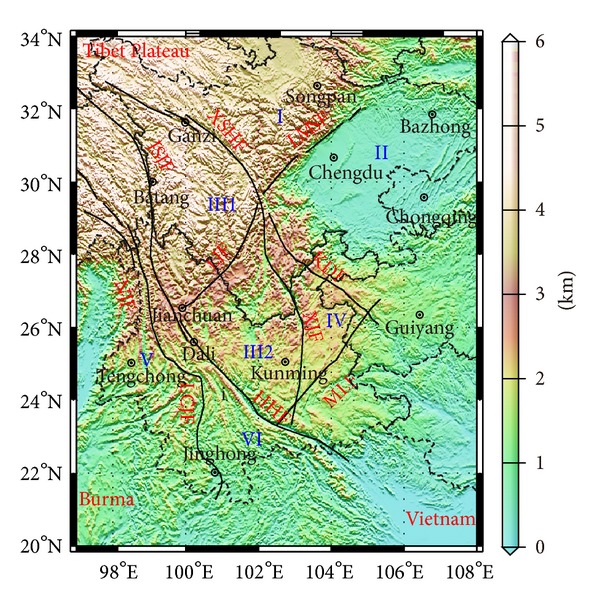
Schematic tectonic map of faults and blocks in the Sichuan-Yunnan region. Topographical data were added by courtesy of ftp://edcftp.cr.usgs.gov/data/gtopo30/global/.

The Sichuan-Yunnan region lies on the southeastern boundary of Qinghai-Tibet Plateau, where the Eurasia plate converges and interacts with the Indian plate. Special tectonic setting makes it one of the most remarkable regions, where the faults are developed and the geological structure is very complicated [16, 17]. In this region, there are Longmen Mountain fault belt (LMSF), Lancangjiang fault belt (LCJF), Lijiang-Xiaojinhe fault belt (LJF), Kangding-Yiliang-Shuicheng fault belt (KDF), Mile-Shizong-Shuicheng fault belt (MLF), and belts that encircle the Sichuan-Yunnan rhombic block, such as Xianshuihe fault belt (XSHF), Anninghe-Xiaojiang fault belt (XJF), Jinshajiang fault belt (JSJF), and Red River fault belt (HHF). These faults divide the crust into multiple blocks (Figure 1). And (I) represents Songpan-Ganzi block (Longmen Mountain block); (II) represents Sichuan Basin (Yangtze block); (III1) represents Sichuan-Yunnan rhombic block (northwestern Sichuan block); (III2) represents Sichuan-Yunnan rhombic block (central Yunnan block); (IV) represents eastern Yunnan block; (V) represents western Yunnan block; (VI) represents Indochina block. Among them are both stable Sichuan Basin and orogenic belts that formed in Cenozoic Era, like Longmen Mountain, northwestern Sichuan and Sanjiang region, and special geotectonic settings such as rift basins of the central Yunnan. Studying characteristics of the crustal field distribution in this region would have very important implications for realizing distribution features of crustal anomalies in basins, orogenic belts, and fracture zones and exploring the connection between the crustal anomaly and the geological structure.

## 3. Calculation of the Crustal Magnetic Field

According to the theory of geomagnetic field potential function, geomagnetic potential can be expressed as spherical harmonic series:

(1) U(r,θ,λ,t) =a∑n=1N∑m=0n(ar)n+1 ×(gnmcos⁡mλ+hnmsinmλ)Pnm(cos⁡θ),

where *λ* and *θ* are longitude and colatitude, respectively, *a* is the Earth's radius (6,371.2 km), *r* is geocentric distance, *P*_*n*_^*m*^(cos⁡*θ*) is Schmidt quasinormalized associated Legendre function of order *n* and degree *m*, and *g*_*n*_^*m*^ and *h*_*n*_^*m*^ are spherical harmonic coefficients of geomagnetic potential. Taking the derivative of magnetic potential function in spherical coordinate system, one can obtain three rectangular coordinate components (*X* = ∂*U*/*r*∂*θ*, *Y* = −∂*U*/*r*sin*θ*∂*λ*, and *Z* = ∂*U*/∂*r*) of the geomagnetic field, namely, *X* (in north direction), *Y* (in east direction), and *Z* (in vertical direction). For harmonic degrees *n* ≥ 16, the northern, eastern, and vertical components of the lithospheric field (Δ*X*, Δ*Y*, and Δ*Z*) can be calculated directly, which can be expressed as follows [18]:

(2) ΔX=∑n=16N∑m=0n(ar)n+2 ×[gnmcos⁡(mλ)+hnmsin(mλ)]∂Pnm(cos⁡θ)∂θ,ΔY=∑n=16N∑m=0n(ar)n+2msinθ ×[gnmsin(mλ)−hnmcos⁡(mλ)]Pnm(cos⁡θ),ΔZ=−∑n=16N∑m=0n(n+1)(ar)n+2 ×[gnmcos⁡(mλ)+hnmsin(mλ)]Pnm(cos⁡θ).

Taking the derivative of each field component with respect to the geocentric distance *r* yields the vertical gradient of the crustal magnetic field [19, 20]. Longitude of our research region extends from 97°E to 108°E and the latitude extends from 21°N to 34°N, which was divided into evenly spaced grid cells with the dimension of 0.1° × 0.1°. Besides Yunnan and Sichuan regions, our target regions cover parts of Guizhou, Guangxi, and Tibet, including also parts of North Vietnam and Burma.

## 4. Basic Features of the Crustal Magnetic Anomaly in Sichuan-Yunnan Region

### 4.1. Features of Magnetic Anomaly Distribution at the Earth's Surface

**Figure 2:**
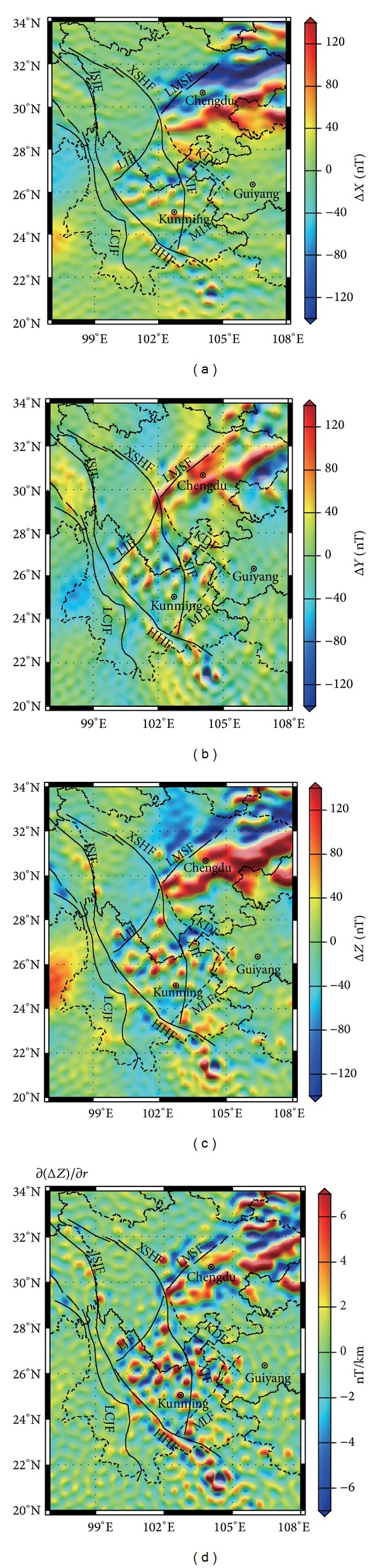
Distribution of the crustal magnetic anomaly of (a) Δ*X*, (b) Δ*Y*, (c) Δ*Z*, and (d) ∂(Δ*Z*)/∂*r*  at the Earth's surface.

Based on the NGDC-720-V3 model, Figure 2 shows the map of magnetic anomaly of Δ*X*, Δ*Y*, and Δ*Z* at the Earth's surface. As we can see, there are large differences in the shape, strike, and intensity of the anomaly for each component. In the Sichuan Basin and its northern part, the strikes are nearly east-west direction for Δ*X*, and north-south for Δ*Y*, and north-east for Δ*Z*. As the scope of positive and negative anomalies is concerned, the anomalies of Longmen Mountain fault are negative for Δ*X* and positive for Δ*Y*. But, for the component Δ*Z*, this faultis located in the transitional region of positive and negative anomalies. The focus and intensity of all components are different from each other. Taking the Sichuan Basin with positive anomaly for example, the positions and intensities of the largest foci of anomalies (component, longitude, latitude, intensity) are (Δ*Z*, 106.2°E, 30.7°N, 461.6 nT; Δ*X*, 108.0°E, 29.6°N, 207.1 nT; Δ*Y*, 106.5°E, 31.5°N, 181.4 nT), respectively. As Δ*Z* has the largest intensity, we focus on the features of Δ*Z* and its vertical gradient ∂(Δ*Z*)/∂*r*.

For the distribution pattern, the magnetic anomaly of Sichuan-Yunnan region obviously has subarea characteristics. Positive anomalies of Sichuan Basin and negative anomalies of Longmen Mountain are widespread and show patches of distribution, whose patterns are relatively simple. The Sichuan-Yunnan rhombic block is divided into the northwestern Sichuan block and the central Yunnan block by the Lijiang-Xiaojinhe faults. The anomaly of central Yunnan block presents ball-like or band-like shape. Positive and negative anomalies appear alternatively, and their distribution patterns are complicated. Anomalies in northwestern Sichuan and western Yunnan are relatively weak, and they are mainly negative. In addition, there are stripy negative and positive anomalies localized along the vicinity of the Red River fault and then extending southeast to the northern part of Vietnam (Hechi area).

Anomalies of the vertical gradient ∂(Δ*Z*)/∂*r* and Δ*Z* (Figure 2(b)) are basically the same in terms of distribution and strike. But compared with Δ*Z*, where the vertical gradient anomalies are strong, the distribution scope of ∂(Δ*Z*)/∂*r* is becoming smaller significantly. Areas of anomalies are increasing, and boundaries of positive and negative anomalies are clearer. In the positive anomaly zone of Sichuan Basin and the negative anomaly zone of Longmen Mountains with widespread and strong magnetic anomalies, the vertical gradient (∂(Δ*Z*)/∂*r*) does not show any strong anomalies over large areas but appears to show banded distribution. We can see clearly that the anomalies in the central Yunnan block are in clustered distribution. Along the Jinshajiang-Red River fault belt, the negative and positive anomaly belts of the vertical gradient show distinct bead-shaped distribution feature.

In order to realize features of anomaly intensity quantitatively, Table 1 lists the principal foci and intensities of Δ*Z* and ∂(Δ*Z*)/∂*r* at the Earth's surface. There are numerous magnetic anomaly foci in the central Yunnan block, but Table 1 shows only two points with the strongest positive and negative anomalies. As demonstrated by Table 1, anomalies of Sichuan-Yunnan region are not uniformly distributed. The anomaly in Sichuan Basin is the strongest, and that of the southeast part of the Red River fault is the second. However, anomalies in the northwestern Sichuan and central Yunnan block are relatively weak.

Later on, the magnetic anomaly intensity is not definitely proportional to variations of the vertical gradient, as only absolute values are concerned. For instance, the strongest anomaly in Sichuan Basin is 461.6 nT, and its vertical gradient value is 13.6 nT/km. In the southeast Red River fault, positive and negative anomalies of the foci are 218.1 nT and −256.7 nT, respectively. The former is corresponding to the vertical gradient value of 16.2 nT/km, but the latter is up to −21.6 nT/km.

The vertical gradient values are not simply related with the rock magnetism but also associated with both the distribution and depths of the magnetic source body. The deeper and more uniformly distributed the magnetic source body is, the smaller the vertical gradient values are. However, at the place where the magnetic source body is shallow and nonuniformly distributed, the vertical gradient value changes greatly. Though Sichuan Basin has the strongest anomaly, its gradient value is relatively small, which means that the anomaly is decreasing slowly, and the buried depth of the magnetic source body is deeper. However, the southeast Red River fault has a large gradient anomaly, indicating a fast decaying anomaly and a shallower magnetic source body depth.

### 4.2. Magnetic Anomaly Decaying with the Altitude

**Figure 3:**
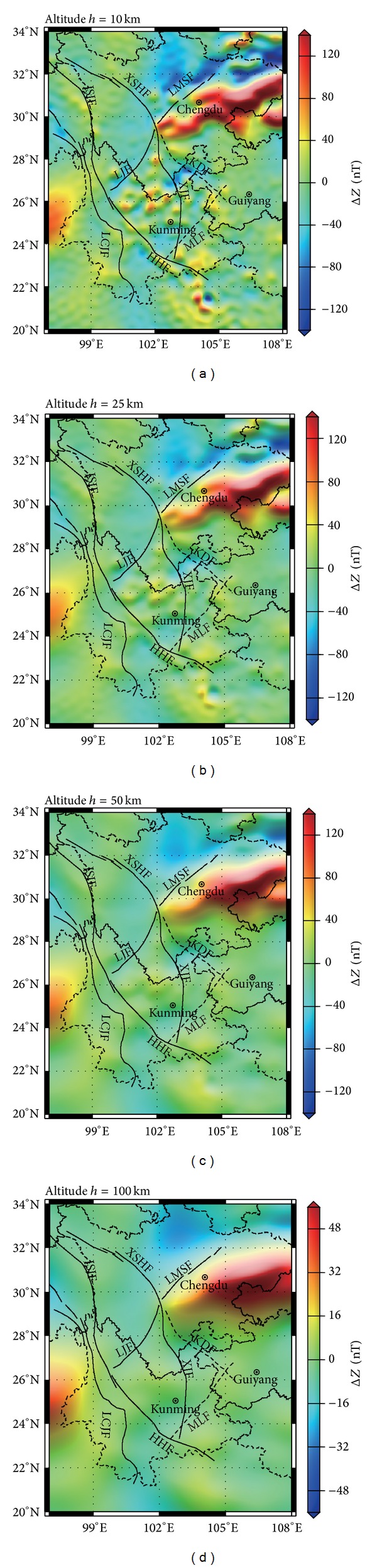
Distribution of the crustal magnetic anomaly at different altitudes: (a) 10 km; (b) 25 km; (c) 50 km; and (d) 100 km.

The decaying speed of the crustal field is directly related to the buried depth and the length scale of the magnetic source body. It can give a better understanding of the deep and shallow structural features of magnetic anomaly sources by calculating magnetic anomaly distribution and analyzing the decaying features at different altitudes above the ground. Thus we calculate the magnetic anomaly distribution of Δ*Z* at various altitudes from ground surface to 400 km (Figure 3). But the distribution patterns of anomalies at 100–400 km altitudes are basically the same; that is, anomalies are positive in Sichuan Basin and negative or weak in other places, so Figure 3 shows only distributions of altitudes 10 km, 25 km, 50 km, and 100 km. It is found that distribution patterns are more and more simple with altitudes increasing, whereas the decaying speed differs drastically in different areas.

Positive anomaly zone of Sichuan Basin always exits from the ground surface to the 100 km altitude. With the altitude increasing, it gradually evolves into an oval-shaped anomaly in a near west-east direction, which agrees with the basin structure. The focus is always staying at the position 106.2°E, 30.2°N nearby. At the altitude 100 km, the intensity of the focus is 74.1 nT. It suggests that the magnetic structure of the Sichuan Basin is relatively stable from the basement to the ground surface.

Anomalies at various altitudes are all negative in the Longmen Mountains block. The distribution scope gradually enlarges with altitude increasing. Directions of anomalies are nearly west-east in the deep layer and east-south in the shallow layer. And the focus position has a trend of moving westward. The positions of the focus are (105.9°E, 32.0°N) on the ground and (102.9°E, 32.4°N) at altitude 100 km, and the intensity is 27.2 nT. The center of this focus moves 3° to the west. Therefore, we can infer that the magnetic source body caused by a negative anomaly is migrating eastward from the deep crust to the surface, reflecting the inconsistency of deep and shallow magnetic body structure in this area. This result is in good agreement with the inference that the Eurasia plate collides with the Indian plate which makes substances inside the Qinghai-Tibet Plateau move eastward [21, 22].

Magnetic anomalies decay very fast in the central Yunnan block. Regional and local magnetic anomaly bands and clumps distributed above ground almost vanish at 25 km altitude, indicating that they are caused by the magnetic substances in the shallow crust. At 50 km altitude, negative anomalies of the Sichuan-Yunnan rhombic block and Longmen Mountain block connect each other. This indicates that they have the same physical nature, that is, weak magnetic substances, in the deep crust. The central Yunnan block anomaly is a comprehensive reflection of the negative anomaly background from the deep within the Earth superposed by local anomalies in the shallow crust.

### 4.3. Distribution Feature of Magnetic Anomalies of Different Wavelength Bands

Estimated from the relation between the geomagnetic harmonic degree and the spatial wavelength (*L* = 2*πa*/*N* provides the minimum wavelength *L* of the cut-off level of harmonic waves degree *N*; *a* = 6371.2 km is the Earth's radius), the crustal field at harmonic degrees 16~720 is corresponding to a wavelength range of 2500 km to 56 km, which reflects a broad range of changes of spatial scales of the field source. Following the Lowes-Mauersberger method [23], the power spectrum of the geomagnetic field is expressed as

(3) $$\begin{array}{c} W\left(n\right)=\left(n+1\right)\overset{n}{\underset{m=0}{\sum }}\left[{\left(g_{n}^{m}\right)}^{2}+{\left(h_{n}^{m}\right)}^{2}\right]. \end{array}$$

**Figure 4:**
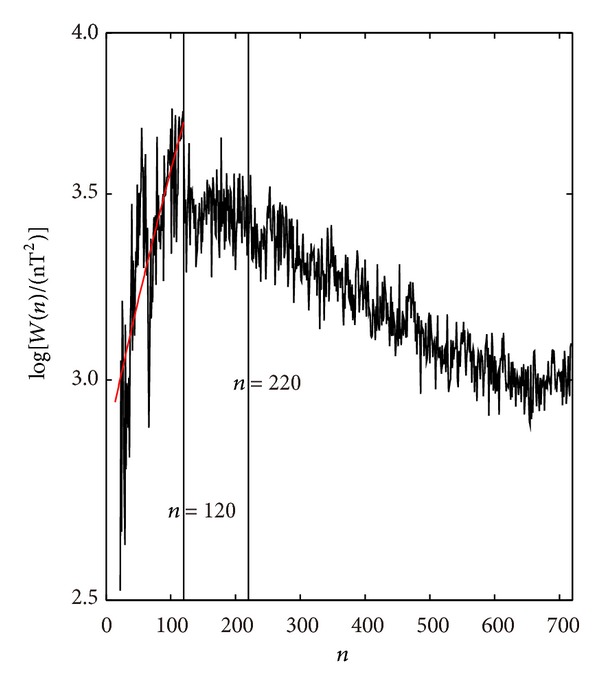
Spherical geomagnetic energy spectra of the NGDC-720-V3 model with the harmonic order *n*.

**Figure 5:**
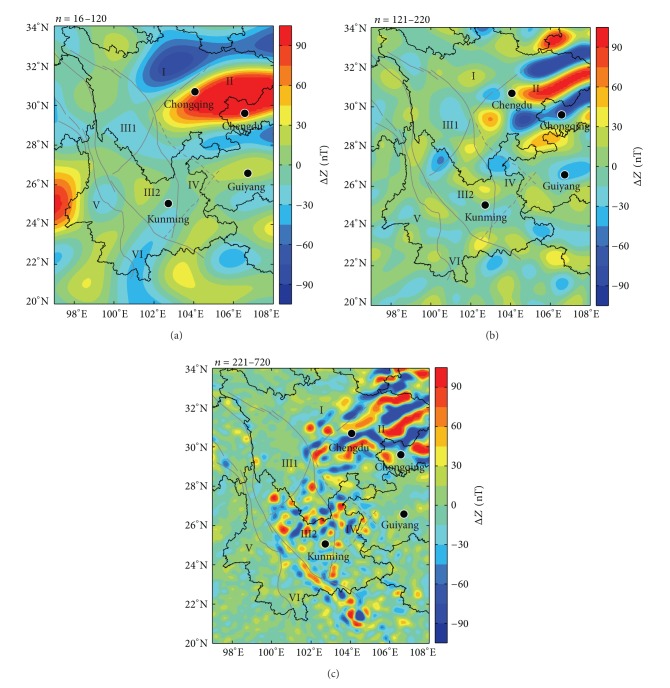
The magnetic anomaly at different wavelength bands: (a) *n* = 16~120; (b) *n* = 121~220; and (c) *n* = 221~720 in Sichuan-Yunnan region.

The variations of crustal magnetic field energy spectra in the NGDC-720-V3 model with harmonic degrees and orders are not linear, calculated according to the above formula. However, variations are fluctuated and meanwhile undergoing a process of being increasing, steady, and decreasing [24]. As shown in Figure 4, the spectra at degrees 16~120 are increased with the increasing harmonic degrees and orders. The variation of spectra at degrees 121~220 is relatively stable. The spectra at degrees 221~720 are gradually decreased. This kind of trend indicates that the harmonic components can be divided into several wavelength bands. The one increasing with the harmonic degrees and orders is known as the long wavelength band (*n* = 16~120), the stable part is known as the middle wavelength band (*n* = 121~220), and the one that seems to decrease linearly is known as the short wavelength band (*n* = 221~720). They are reflecting magnetic features from the deep crust, the middle section, and the shallow layer. Distributions of three wavelength bands of the Δ*Z* anomaly at the Earth's surface are shown in Figures 5(a), 5(b), and 5(c).

As it is shown from Figure 5, the distribution of the short wavelength anomaly agrees with that of the vertical gradient ∂(Δ*Z*)/∂*r*. There are several negative and positive anomalies bands in Sichuan Basin and Longmen Mountain areas and clumps of anomalies in the central Yunnan area. Strips of positive and negative anomalies can be clearly seen along the Ailao Mountain-Red River fault. Round anomalies in the central Yunnan area and band-like ones on both sides of the Red River fault only appear in the short wavelength band, which further indicates that they are caused by the rock magnetism of the shallow crust.

Areas of positive and negative anomalies of the middle wavelength band are increasing. However, positive anomalies of Sichuan Basin and negative anomalies on its south and north sides are stronger, and anomalies in other areas are weaker. The negative anomaly in the southern part of Sichuan Basin is approximately in west-east direction, which has a clear interaction angle with the anomaly of the short wavelength band, indicating that the magnetic structure is inconsistent from the deep to the shallow layers in this area.

Patterns of anomalies of the long wavelength band are simple. The positive anomaly of Sichuan Basin has elliptical shape in nearly west-east direction, encircled by negative anomalies. This implies that the magnetic structure of the deep interior in Sichuan Basin differs much with that of its surrounding areas. Focal positions of positive anomalies of three wavelength bands are basically the same in the Sichuan Basin, which are always near the position of 106.2°E, 30.2°N, reflecting that they have stable positions of magnetic source body.

### 4.4. Relation between the Magnetic Anomaly and Geological Structure

Comparing the corresponding relationship between the magnetic anomaly and the geological structure at different altitudes and in different wavelength bands, we can find out the relation between the anomaly and the crustal blocks and faults.

(1) A close relationship is available between the magnetic anomaly and crustal blocks. As we can see from the distribution of Δ*Z*, the positive anomaly is distributed in ancient blocks of stable structure (Sichuan Basin); negative and weak anomalies are in orogenic belts with intense tectonic activities, and anomalies are complex in fracture belts. The positive anomaly in Sichuan Basin is strong and widely spread; its focal intensity is three times that of the central Yunnan area. There are strong and negative anomalies in the Longmen Mountain block with intense tectonic activities. Anomalies in northwestern Sichuan and western Yunnan are weaker, and the maximum strength is not more than 80 nT. Several bead-like rifted basins are developed in the central Yunnan block [25], whose anomalies are round, linear, and bead-like. And positive anomalies alternate with negative ones; their distributions are complicated.

(2) The corresponding relationship of magnetic anomaly and faults is determined. As we can see from distributions of Δ*Z*, ∂(Δ*Z*)/∂*r* (Figure 2) and short wavelength band at degrees *n* ≥ 221 (Figure 5(c)), several deep seated fault zones are the positive and negative anomalies boundary or transitional zones of strong and weak anomalies. The Longmen Mountain fault is the positive and negative boundary of Sichuan Basin and Longmen Mountain block. The Sichuan-Yunnan rhombic block is divided into the northwestern Sichuan block and the central Yunnan block by the Lijiang-Xiaojinhe faults which are transitional zones of strong and weak anomalies. The anomaly in the northwestern Sichuan block is weaker, and clumps of anomalies in the central Yunnan block are relatively stronger. Anomalies on west and east sides of the Red River fault have obvious differences: the one on the west side is weak and the other on the east side is stronger. The above characteristics of distribution of strong and weak anomalies on both sides of the faults support the inference that the border between the Qinghai-Tibet Plateau and Yangtze block is divided by the Longmen Mountain fault, Lijiang-Xiaojinhe faults, and the Red River fault [26]. We can also find out that, from Figure 2, no significant changes were found in anomalies on both sides of the Xianshuihe fault, Jinshajiang fault, and Lancang Jiang fault, indicating that there are no obvious differences in magnetic materials on two sides of these faults.

It is worth noting that available research shows that the Red River fault is the boundary of the Eurasia plate and the India-Australia plate, whose east side extends into the South China Sea [27, 28]. However, the magnetic anomaly band along the Red River fault stops in the northern part of Vietnam and does not extend eastward to the South China Sea. This characteristic of the relation between the magnetic anomaly and the strike of the Red River fault still needs further investigation. In addition, based on the seismic active tectonics division, the eastern boundary of the Sichuan-Yunnan rhombic block is boarded by the Anninghe-Zemuhe-Xiaojiang faults [29, 30]. But we can see from distributions of Δ*Z*, ∂(Δ*Z*)/∂*r* in Figure 2 that there is no significant boundary on both sides of the Xiaojiang fault. Areas of stronger anomalies in the central Yunnan block extend to the east of the Xiaojiang fault, which is roughly boarded by the Kangding-Yiliang-Shuicheng faults and the Mile-Shizong-Shuicheng faults (marked by the dash line in Figure 1). Geological research shows that many features of strata, tectonics, and mineral deposits are similar on both sides of the Xiaojiang fault [31]. Thus, from the point of view of the magnetic anomaly, the eastern boundary of the Sichuan-Yunnan rhombic block should be divided by the Kangding-Yiliang-Shuicheng faults and the Mile-Shizong-Shuicheng faults.

(3) Crustal structures revealed by the anomaly and the geophysical fields are analyzed comparatively. The gravitational anomaly and seismic wave detections suggest that there are larger lateral variations of the crustal structure in Sichuan-Yunnan region. The crust of the Earth can be divided into upper and lower layers. There are no large variations in the upper crust. Most of them lie between 10 km and 15 km, and crustal variations are mainly caused by the lower crust. Generally, the Moho depth is gradually increasing from southwest to northwest in the Sichuan-Yunnan region [32–35]. Average depths of the Moho are about 38 km in the south Red River fault, 45 km in Sichuan Basin, and 56 km in northwestern Sichuan. The average thickness of the Songpan-Ganzi block (Longmen Mountain block) is up to 60–62 km. However, this kind of variation is not available for the crustal anomaly, which means that there is no direct connection between the crustal anomaly and the Moho depth.

Though physical properties of the crust medium reflected by the crustal and gravitational anomalies are different, there is no comparability in the distribution patterns of both. However, the crust density inhomogeneity obtained by gravity anomaly inversion [33] shows a better corresponding relationship with positive and negative or strong and weak anomalies. The density is relatively higher in Sichuan Basin, where the anomaly is positive, but densities are comparatively lower in the Longmen Mountain block, western Sichuan block, and an area west of Red River fault, whose anomalies are negative or weak. High and low densities distribute in interval in the central Yunnan block where anomalies are round and band-like, alternating with positive and negative distribution. The Longmen Mountain fault and Lijiang-Xiaojinhe faults are the dense gravity anomaly gradient belts and boundary areas of strong and weak anomalies. Along the Red River fault there are a series of regional high and low density anomalies nearby, while bead-like anomalies are also distributed along this fault.

Seismic wave velocity structure research shows that the crustal wave velocity in the Sichuan-Yunnan area has great changes. In the upper crust, a positive anomaly velocity zone exists in the Sichuan Basin, whereas a negative anomaly velocity zone exists in the western Sichuan Plateau and western Yunnan. The boundary between positive and negative anomaly zones is the Longmen Mountain fault zone, and the Red River fault is the transitional area of positive and negative anomalies [36, 37]. There is a corresponding relationship of the seismic wave velocity anomaly and the crustal anomaly. That is, where the magnetic anomaly is positive, the seismic wave velocity is positive, and where the magnetic anomaly is negative or weak, the seismic wave velocity is negative.

Geothermal field observation shows that the lateral variation of the geothermal field has significant change in the Sichuan-Yunnan area, and high geothermal characteristic is available in western orogenic belt [38–43]. The average crustal heat flow value is 61 mWm^−2^ in China, while the values are 72 mWm^−2^ in western Sichuan area and 82 mWm^−2^ in western Yunnan area. Status of low temperature appears in the Sichuan Basin, and the average heat flow value is 53 mWm^−2^. Terrestrial heat flow values on the west side of the Red River fault are higher, ranging 60~80 mWm^−2^, but values on the east side of the fault are lower, ranging 50~60 mWm^−2^. High geothermal feature is available in the Yunnan area, and the distribution of the geothermal field has the trend of the west being higher than the east and gradually descends from west to east, like a wave. In the Sichuan-Yunnan region, the main reason is the subduction and extrusion of the Indian plate. In Yunnan and Panxi regions, there is a phenomenon of high heat flow value [43], which is usually connected with tectonic activities (such as the magmatism). Comparing the distributions of the anomaly and the terrestrial heat flow, we can find out that weak or negative anomalies values are corresponding to areas of the high terrestrial heat flow value, but the positive anomaly is in line with the low terrestrial heat flow value.

(4) Crustal anomaly distributions have tectonic and geodynamic significances. From the plate tectonics point of view, due to the collision of India and Eurasia plates, the Qinghai-Tibet Plateau is blocked by the Siberian platform in the north. Results under this kind of tectonic framework show that, on the one hand, the plateau absorbs the north-south strong compression through the crust thickening and shortening; on the other hand, it seeks a channel that the plateau materials escape to east and west [37, 44]. However, no matter what the escape way it is, the Sichuan-Yunnan region in the southeastern Tibetan plateau is the principal access where the plateau materials flow east. Prevented by the high-strength block (i.e., the Sichuan Basin), east flowing materials split into two of the northeast and southeast parts. This dynamic process is verified in sideways by strike features of anomaly distributions. Take the Longmen Mountain area to the north of Sichuan Basin for example; the vertical gradient anomaly reflecting the mid-upper crust magnetic source body anomaly shows a distinct east-north trend, which agrees with the geological structure strike. Anomalies are weaker in Cenozoic orogenic belts such as the western Sichuan and Sanjiang region. Meanwhile, there are several rifted basins in the south of the Sichuan-Yunnan rhombic block (e.g., the central Yunnan block), where positive and negative anomalies are band-like and round.

## 5. Discussions and Conclusions

As the NGDC-720 model has been compiled from satellite, shipborne, and aeromagnetic measurements, the ground survey especially, the input data has already included the information of geological structure. The relation between crustal anomaly and geological structure could be reflected by the model which provides the lithospheric magnetic field vector at any desired location and altitude close to and above the Earth's surface. Thus, this model is also a useful tool in geological and tectonic studies of the lithospheres.

(1) The crustal magnetic anomaly given by the NGDC-720 geomagnetic model has distinct local features in Sichuan-Yunnan area. A positive anomaly is strong in the Sichuan Basin with steady tectonics, but a negative anomaly is stronger in Longmen Mountain block with intense tectonic activities. Anomalies of the central Yunnan block with complex structures are round, linear, and bead-like, and positive and negative anomalies distribute alternatively.

(2) The crustal anomaly has close relation with the geological structure. Anomalies in the northwestern Sichuan, western Yunnan orogenic belts, and south of the Red River fault are relatively weak. The Longmen Mountain fault, Lijiang-Xiaojinhe, and Red River fault belts are the transitional zones of strong and weak (or negative and positive) anomalies. The eastern boundary of anomalies in the central Yunnan block is bordered by the Kangding-Yiliang-Shuicheng and Mile-Shizong-Shuicheng fault belts.

(3) Distributions of magnetic anomalies at different altitudes and of different wavelength bands show that the magnetic structure of the Sichuan Basin is relatively steady from the deep interior of the crust to the ground surface. In the Longmen Mountains area with negative anomaly, the position of the magnetic source body gradually moves eastward from the deep crust to the shallow part. In the central Yunnan block, the magnetic field source of the anomaly is shallow.

(4) The crustal magnetic anomaly has corresponding relationship with geophysical fields. Where the magnetic anomaly is positive, the seismic wave velocity is positive, the crust density is relatively higher, corresponding to areas of the high terrestrial heat flow values; and where the magnetic anomaly is negative or weak, the seismic wave velocity is negative, the density is comparatively lower, and the terrestrial heat flow value is low. The dense gravity anomaly gradient belt is the boundary area of strong and weak anomalies.

These results imply that the physical link between the magnetic anomalies and geological structures is not simple and straightforward. And, in Sichuan-Yunnan region, magnetic anomalies have close but complicated relations with the crust and upper mantle.

## Figures and Tables

**Table 1:** The location and intensity of magnetic anomaly foci of Δ*Z* and ∂(Δ*Z*)/∂*r* for each block at the Earth's surface.

Region	ΔZ			∂(Δ*Z*)/∂*r*		
	*λ*	*φ*	Intensity	*λ*	*φ*	Intensity
	/°E	/°N	/nT	/°E	/°N	(nT*·*km^−1^)
Sichuan Basin	106.2	30.7	461.6	106.1	30.7	13.6
Longmen Mountain block	105.9	32	−187.8	105.9	32.1	−10.4
Northwestern Sichuan block	97.7	29.1	−93.6	97.7	29.1	−4.6
Central Yunnan block	102.6 101.8	26.6 25.6	−121.0 120.5	103.5 101.8	27.3 25.6	−7.2 7.9

Southeastern part of Red River fault	104.5 104.0	21.4 21.5	−256.7 218.1	104.5 104.0	21.4 21.5	−21.6 16.2

## References

[1] Xu WY (2009). *Physics of Electromagnetic Phenomena of the Earth*.

[2] Xu WY, Bai CH, Kang GF (2008). Global models of the Earth’s crust magnetic anomalies. *Progress in Geophysics*.

[3] Langlais B, Lesur V, Purucker ME, Connerney JEP, Mandea M (2010). Crustal magnetic fields of terrestrial planets. *Space Science Reviews*.

[4] Thébault E, Purucker M, Whaler KA, Langlais B, Sabaka TJ (2010). The magnetic field of the earth's lithosphere. *Space Science Reviews*.

[5] Maus S (2008). The geomagnetic power spectrum. *Geophysical Journal International*.

[6] Hemant K, Mitchell A (2009). Magnetic field modelling and interpretation of the Himalayan-Tibetan Plateau and adjoining north Indian Plains. *Tectonophysics*.

[7] Maus S, Yin F, Lühr H (2008). Resolution of direction of oceanic magnetic lineations by the sixth-generation lithospheric magnetic field model from CHAMP satellite magnetic measurements. *Geochemistry, Geophysics, Geosystems*.

[8] Olsen N, Mandea M, Sabaka TJ, Tøffner-Clausen L (2009). CHAOS-2—a geomagnetic field model derived from one decade of continuous satellite data. *Geophysical Journal International*.

[9] Olsen N, Lühr H, Sabaka TJ (2006). CHAOS-a model of the Earth's magnetic field derived from CHAMP, Ørsted, and SAC-C magnetic satellite data. *Geophysical Journal International*.

[10] Sabaka TJ, Olsen N, Purucker ME (2004). Extending comprehensive models of the Earth’s magnetic field with Ørsted and CHAMP data. *Geophysical Journal International*.

[11] Maus S, Barckhausen U, Berkenbosch H (2009). EMAG2: a 2–arc min resolution Earth Magnetic Anomaly Grid compiled from satellite, airborne, and marine magnetic measurements. *Geochemistry, Geophysics, Geosystems*.

[12] Hamoudi M, Thébault E, Lesur V, Mandea M, Thébault E, GeoforschungsZentrum Anomaly Magnetic Map (GAMMA): a candidate model for the world digital magnetic anomaly map (2007). *Geochemistry, Geophysics, Geosystems*.

[13] Lesur V, Wardinski I, Rother M, Mandea M (2008). GRIMM: the GFZ Reference Internal Magnetic Model based on vector satellite and observatory data. *Geophysical Journal International*.

[14] Hemant K, Thébault E, Mandea M, Ravat D, Maus S (2007). Magnetic anomaly map of the world: merging satellite, airborne, marine and ground-based magnetic data sets. *Earth and Planetary Science Letters*.

[15] Maus S (2010). An ellipsoidal harmonic representation of Earth’s lithospheric magnetic field to degree and order 720. *Geochemistry, Geophysics, Geosystems*.

[16] Xu XW, Wen XZ, Zheng RZ, Ma W, Song F, Yu G (2003). Pattern of latest tectonic motion and its dynamics for active blocks in Sichuan-Yunnan region. *Science in China D: Earth Sciences*.

[17] Su YJ, Qin JZ (2001). Strong earthquake activity and relation to regional neotectonic movement in Sichuan-Yunnan region. *Earthquake Research China*.

[18] Kang GF, Gao GM, Bai CH, Shao D, Feng LL (2012). Characteristics of the crustal magnetic anomaly and regional tectonics in the Qinghai-Tibet Plateau and the adjacent areas. *Science China Earth Sciences*.

[19] Gao GM, Kang GF, Bai CH (2012). Characteristics of the spatial distribution and the secular variation of the main geomagnetic field gradients. *Chinese Journal of Geophysics*.

[20] An ZC, Wang YH, Xu YF (1991). Calculations and analyses of vertical gradient of the geomagnetic field in China and adjacent areas. *Chinese Journal of Space Science*.

[21] Molnar P, Lyon-Caen H (1989). Fault plane solutions of earthquakes and active tectonics of the Tibetan Plateau and its margins. *Geophysical Journal International*.

[22] Qiao XJ, Wang Q, Du RL (2004). Characteristics of current crustal deformation of active blocks in the Sichuan-Yunnan region. *Chinese Journal of Geophysics*.

[23] Lowes FJ (1974). Spatial power spectrum of the main geomagnetic field and extrapolation to the core. *Geophysical Journal of the Royal Astronomical Society*.

[24] Kang GF, Gao GM, Bai CH, Wang J, Shao D (2010). Distribution of the magnetic anomaly for the CHAMP satellite in China and adjacent areas. *Chinese Journal of Geophysics*.

[25] Qiu RZ, Li TD, Deng JF (2006). A new pattern of tectonic units of China considered in light of the lithosphere. *Geology in China*.

[26] Zhong K, Xu MJ, Wang LS (2005). Study on characteristics of gravity field and crustal deformation in Sichuan-Yunnan region. *Geological Journal of China Universities*.

[27] Liu BM, Xia B, Li XX (2006). Southeastern extension of the Red River fault zone (RRFZ) and its tectonic evolution significance in Western South China Sea. *Science in China D: Earth Sciences*.

[28] Xu Y, Liu JH, Liu FT, Song H, Hao T, Jiang W (2005). Crust and upper mantle structure of the Ailao Shan-Red River fault zone and adjacent regions. *Science in China D: Earth Sciences*.

[29] Cui JW, Zhang XW, Tang ZM (2006). Tectonic divisions of the Qinghai-Tibet Plateau and structural characteristics of deformation on their boundaries. *Geology in China*.

[30] Yi GX, Wen XZ, Su YJ (2008). Study on the potential strong-earthquake risk for the eastern boundary of the sichuan-yunnan active faulted-block, China. *Chinese Journal of Geophysics*.

[31] Wang BL, Lu SK, Hu JG (2004). A tentative description of the Chuan-Dian-Qian rhombic block. *Yunnan Geology*.

[32] Zhong K, Xu MJ, Wang LS (2005). Study on continetal deformation features in Sichan-Yunnan region from aeromagnetic and gravity data. *Advances in Earth Science*.

[33] Lou H, Wang C (2005). Wavelet analysis and interpretation of gravity data in Sichuan-Yunnan region, China. *Acta Seismologica Sinica*.

[34] Zhang PZ (2013). A review on active tectonics and deep crustal processes of the Western Sichuan region, eastern margin of the Tibetan Plateau. *Tectonophysics*.

[35] Zhang Z, Chen Y, Li F (2008). Reconstruction of the S-wave velocity structure of crust and mantle from seismic surface wave dispersion in Sichuan-Yunnan Region. *Chinese Journal of Geophysics*.

[36] Wang C, Mooney WD, Wang X, Wu J, Lou H, Wang F (2002). Study on 3-D velocity structure of crust and upper mantle in Sichuan-Yunnan region, China. *Acta Seismologica Sinica*.

[37] Huang JL, Song XD, Wang SY (2003). Fine structure of Pn velocity beneath Sichuan-Yunnan region. *Science in China D: Earth Sciences*.

[38] An MJ, Shi YL (2007). Three-dimensional thermal structure of the Chinese continental crust and upper mantle. *Science in China D: Earth Sciences*.

[39] Wang Y, Deng JF, Wang JY, Xiong LP (2001). Terrestrial heat flow pattern and thermo-tectonic domains in the continental area of China. *Journal of the Graduate School of the Chinese Academy of Sciences*.

[40] Wang Y (2001). Heat flow pattern and lateral variations of lithosphere strength in China mainland: constraints on active deformation. *Physics of the Earth and Planetary Interiors*.

[41] Hu SB, He LJ, Wang JY (2001). Compilation of heat flow data in the China continental area (3rd edition). *Chinese Journal of Geophysics*.

[42] Wang Y, Wang JY, Xiong LP, Deng JF (2001). Lithospheric geothermics of major geotectonic units in China mainland. *Acta Geoscientia Sinica*.

[43] Wu QF, Zu JH, Xie YZ, Wang D (1988). Characteristics of the geotherm in Yunnan region. *Seismology and Geology*.

[44] Schoenbohm LM, Burchfiel BC, Chen LZ (2006). Propagation of surface uplift, lower crustal flow, and Cenozoic tectonics of the Southeast margin of the Tibetan Plateau. *Geology*.

